# Testing for canonical form orientation in speech tempo perception

**DOI:** 10.1177/17470218231198344

**Published:** 2023-09-14

**Authors:** Leendert Plug, Robert Lennon, Rachel Smith

**Affiliations:** 1 University of Leeds, Leeds, UK; 2 University of Glasgow, Glasgow, UK

**Keywords:** Speech tempo, articulation rate, deletion, perception, English

## Abstract

We report on two experiments that aimed to test the hypothesis that English listeners orient to full pronunciation forms—“canonical forms”—in judging the tempo of speech that features deletions. If listeners orient to canonical forms, this should mean that the perceived tempo of speech containing deletions is highly relative to the speech’s articulation rate calculated on the basis of surface phone strings. We used controlled stimuli to test this hypothesis. We created sentences with one ambiguous word form (for example, *support~sport*), to give half of the listeners an orthographic form that includes *support* and the other half an otherwise identical orthographic form with *sport*. In both experiments, listeners judged the tempo of the sentences, which allowed us to assess whether the difference in imposed interpretation had an impact on perceived tempo. Experiment 1 used a tempo rating task in which listeners evaluated the tempo of experimental stimuli relative to comparison stimuli, on a continuous scale. Experiment 2 used a tempo comparison task in which listeners judged whether second members of stimulus pairs were slower or faster than first members. Both experiments revealed the predicted effect of the imposed word interpretation: sentences with an imposed “schwa” interpretation for the ambiguous word form were judged faster than (the same) sentences with an imposed “no schwa” interpretation. However, in both experiments the effect was small and variables related to the experimental design had significant effects on responses. We discuss the results’ implications for our understanding of speech tempo perception.

## Introduction

Phonetic reduction phenomena, including phone and syllable deletion, are ubiquitous in normal speech (Bürki, 2018; Ernestus, 2014; Johnson, 2004; Warner et al., 2022). While “[t]he comprehension of reduced speech is a complex process of which we have just started to discover the general mechanisms” (Ernestus, 2014, p. 36), there is considerable evidence to support the notion that full pronunciation forms—“canonical forms”—have a different status from reduced forms in speech perception. For example, full pronunciation forms tend to be recognised more quickly than moderately reduced forms (Janse et al., 2007; Ranbom & Connine, 2007) and are shadowed more efficiently (Brouwer et al., 2010), even if the latter are more frequent in normal speech. Full pronunciation forms also form the basis of listeners’ phonotactic generalisations despite the ubiquity of reduction (Bürki, 2018).

Moreover, there is evidence of what we will call “canonical form orientation” in speech processing: listeners accessing and deriving information from full pronunciation forms even when processing reduced speech. For example, listeners may report hearing phonemes that are absent from the signal due to assimilation or other reduction processes (Kemps et al., 2004; Mitterer & Blomert, 2003; Mitterer et al., 2008). This phenomenon can be seen as an extension of the “phoneme restoration effect,” in which listeners report hearing phonemes that are masked by speech-like noise (Mattys et al., 2014; Warren & Obusek, 1971). It suggests that the processing of reduced forms involves the activation of their corresponding canonical forms, and that this activation may override bottom-up information regarding the presence or absence of acoustic cues (Kemps et al., 2004).

In this article, we address the impact of deletions on speech tempo perception. Previous studies have provided some support for the notion that listeners orient to canonical forms when judging the tempo of speech containing phone and syllable deletions. Koreman (2006) showed that listeners perceived tempo differences between spontaneously produced German intonation phrases with similar surface but different canonical rates. He selected phrases with reference to their measured phone rates and numbers of phone deletions to make up groups such as *fast~clear* (high canonical rates, few deletions), *fast~sloppy* (high canonical rates, many deletions), *normal~sloppy* (normal canonical rates, many deletions) and *slow~clear* (low canonical rates, few deletions). He ensured that phrases in the *fast~sloppy* and *normal~clear* groups were not significantly different in surface articulation rate, while different in canonical rate due to the deletions in *fast~sloppy* phrases—and the same for *normal~sloppy* and *slow~clear*. Listeners heard pairs of phrases that crossed these groups and judged which pair member was faster. Results showed that they perceived utterances with similar surface but different canonical rates as different in tempo: for example, *fast~sloppy* utterances were perceived as faster than *normal~clear* ones.

Plug et al. (2022) implemented a similar design to Koreman’s using stretches sampled from a corpus of unscripted English speech. They organised the stretches into groups within which either canonical rates varied while surface rates were close to constant, or *vice versa*. They constructed separate groups for syllable rate and phone rate. Like Koreman’s, their listeners perceived tempo differences between utterances with similar surface but different canonical rates, albeit for syllable rates only.

In accounting for his results, Koreman (2006) emphasises listeners’ implicit understanding of the association between high speech tempo and high deletion rates. On this reasoning—which also applies to Plug et al. (2022)—the fact that phone or syllable deletions make speech sound relatively fast does not necessarily mean that listeners map canonical unit counts to signal duration in estimating tempo. Rather, listeners’ judgements may have hinged on their recognition of relatively “casual speech, the register in which reduction is most common” (Ernestus, 2014). This reasoning is supported by Reinisch (2016). In Reinisch’s experiments, listeners judged the tempo of naturally produced normal and fast speech, and speech that results from linear tempo manipulations. A German utterance was produced at normal tempo, with few deletions, and at fast tempo with more deletions. Both were manipulated to create an additional “normal tempo” version with the fast-tempo deletions and a “fast tempo” one without. The four versions were first used as context sentences in an implicit tempo perception task (Bosker, 2017; Mitterer, 2018; Newman & Sawusch, 1996, 2009; Reinisch et al., 2011; Sawusch & Newman, 2000), and then in an explicit tempo perception task involving paired comparison. In the latter task, listeners heard no consistent difference between naturally fast and linearly compressed versions, but in the implicit task, the naturally fast utterance version was perceived as faster than the linearly compressed version. This is not fully explained by orientation to canonical forms, as the canonical rates were the same for the naturally fast and linearly compressed utterance versions. Rather, listeners appeared to draw on their knowledge that phone deletions tend to occur in fast speech.

These results raise the question of whether Koreman’s (2006) and Plug et al.’s (2022) finding that tempo judgements are influenced by canonical forms could be replicated in an experimental design that minimises listeners’ ability to distinguish stimuli in terms of their overall production style. We report on two experiments that address this question. We used the speech of one speaker only, speaking in one style. We also kept tempo manipulations to a minimum by capitalising on the fact that in English, the non-realisation—or non-segmental realisation—of schwa in an unstressed syllable may result in a surface realisation of a legal consonant cluster associated with a different real word than the intended one. For example, “schwa deletion” in *support* and *terrain* results in surface realisations that are highly similar to those of *sport* and *train*, at least at the level of broad phonetic description. This makes it possible to present listeners with such surface realisations and convince some that they are listening to disyllabic words (*support*, *terrain* etc.) and others that they are listening to monosyllabic ones (*sport*, *train* etc.). Asking listeners to judge the tempo of utterances in which these forms are embedded then allows us to test whether the difference in interpretation—which entails a difference in phone and syllable numbers—has an impact on listeners’ tempo judgements. We opted for an explicit tempo judgement task, rather than the type of implicit task used by Reinisch (2016) and others, where implicit perception of utterance rate informs the phonetic categorisation of a segment at sentence end. This choice was because we thought that creating materials with two separate sources of ambiguity could overcomplicate listeners’ task; we return to this point in the General Discussion.

## Experiment 1

### Method

#### Participants

Seventy native speakers of British English (56 females) in the age range 18–35 (*M* = 22) years participated in this experiment. All self-reported as having grown up in a monolingual household and having no known hearing problems. All provided informed written consent in line with institutional ethics clearance (University of Leeds, Faculty of Arts, Humanities and Cultures Ethics Committee, LTSLCS-072). All except six were paid for their time.

#### Materials

##### Sentence creation

To create our stimulus set, we identified the 26 word pairs in Table 1 as potential loci of lexical ambiguity due to schwa deletion. The word pairs have two general initial structures: *fricative*–(*schwa)–plosive* (e.g., *support~sport*), and *plosive–schwa–liquid*, where the plosive is either voiced (e.g., *below~blow*) or voiceless (e.g., *collapse*~*claps*) and. The set includes several subsets of morphologically related pairs, such as *sport~support*, *sported~supported*, *sporting~supporting*, and *sports~supports*.

**Table 1.** Experimental word pairs and sentences, with reference transcriptions for [–schwa] and [+schwa] word pair members.

Structure	Sentence	[–schwa] word form	[+schwa] word form
fricative-(schwa)-plosive	1. He wanted to *sport~support* it.	*sport* /ˈspɔːt/	*support* /səˈpɔːt/
	2. He *sported~supported* that jumper.	*sported* /ˈspɔːtɪd/	*supported* /səˈpɔːtɪd/
	3. He listed *sporting~supporting* laws.	*sporting* /ˈspɔːtɪŋ/	*supporting* /səˈpɔːtɪŋ/
	4. He often *sports~supports* this.	*sports* /ˈspɔːts/	*supports* /səˈpɔːts/
voiced plosive-(schwa)-liquid	5. Why not *blow~below* the candles?	*blow* /ˈbləʊ/	*below* /bəˈləʊ/
	6. They *dried~deride* it.	*dried* /ˈdɹaɪd/	*deride* /dəˈɹaɪd/
	7. The distance was *drivable~derivable*.	*drivable* /ˈdɹaɪvəbl̩/	*derivable* /dəˈɹaɪvəbl̩/
	8. They *drive~derive* it.	*drive* /ˈdɹaɪv/	*derive* /dəˈɹaɪv/
	9. This tool *drives~derives* it.	*drives* /ˈdɹaɪvz/	*derives* /dəˈɹaɪvz/
	10. We are *driving~deriving* the length.	*driving* /ˈdɹaɪvɪŋ/	*deriving* /dəˈɹaɪvɪŋ/
	11. He spotted the *gorilla~griller*.	*griller* /ˈɡɹɪlə/	*gorilla* /ɡəˈɹɪlə/
	12. I counted five *gorillas~grillers*.	*grillers* /ˈɡɹɪləz/	*gorillas* /ɡəˈɹɪləz/
voiceless plosive-(schwa)-liquid	13. He predicted the *claps~collapse*.	*claps* /ˈklaps/	*collapse* /kəˈlaps/
	14. It was *clean~Colleen* today.	*clean* /ˈkliːn/	*Colleen* /kəˈliːn/
	15. It was the first *clone~cologne*.	*clone* /ˈkləʊn/	*cologne* /kəˈləʊn/
	16. I tried these *clones~colognes*.	*clones* /ˈkləʊnz/	*colognes* /kəˈləʊnz/
	17. They *clued~collude* in the police.	*clued* /ˈkluːd/	*collude* /kəˈluːd/
	18. It was that *cream~Kareem* again.	*cream* /ˈkɹiːm/	*Kareem* /kəˈɹiːm/
	19. I love this *cress~caress*.	*cress* /ˈkɹes/	*Caress* /kəˈɹes/
	20. They *crowed~corrode* on the roof.	*crowed* /ˈkɹəʊd/	*corrode* /kəˈɹəʊd/
	21. He spotted the *Kroner~corona*.	*Kroner* /ˈkɹəʊnə/	*corona* /kəˈɹəʊnə/
	22. I counted 10 *Kroners~coronas*.	*Kroners* /ˈkɹəʊnəz/	*coronas* /kəˈɹəʊnəz/
	23. I see John’s *plight~polite*.	*plight* /ˈplaɪt/	*polite* /pəˈlaɪt/
	24. At the end we *parade~prayed*.	*prayed* /ˈpɹeɪd/	*parade* /pəˈɹeɪd/
	25. I don’t like this *train~terrain*.	*train* /ˈtɹeɪn/	*terrain* /təˈɹeɪn/
	26. I saw those *trains~terrains*.	*trains* /ˈtɹeɪnz/	*terrains* /təˈɹeɪnz/

In all pairs, the disyllabic pair member contains a pre-stress schwa. In this position, schwa deletion has been shown to be a gradient process (Davidson, 2006; Patterson et al., 2003). We, therefore, assume that the disyllabic pair member has one canonical form that includes /ə/; see Bürki and Gaskell (2012) for evidence supporting this assumption. The phonemic transcriptions in Table 1 follow the transcription conventions of Collins and Mees (2013). We ensured that our speaker consistently used “weak form” realisations of the initial syllables—hence /dəˈɹaɪv/ rather than /diˈɹaɪv/ for *derive*; /bəˈləʊ/ rather than /biˈləʊ/ for *below* and so on.

As shown in Table 1, for each word pair we constructed a single short carrier sentence in which either pair member was semantically and grammatically fitted. These sentences served as the basis for our experimental stimuli. Moreover, for each lexical pair we identified one additional lexical item that was similar in phonological make-up to the monosyllabic pair member and one that was similar to the disyllabic one. We embedded these (unambiguous) additional items in the same carrier sentences: for example, “He wanted to *start~restart* it.” alongside “He wanted to *sport~support* it.” (Sentence 1). The resulting additional sentences served as comparison and filler sentences in our experimental designs. All comparison and filler sentences are included in the data files that accompany this article.

As the ease of interpretation of sentences may be relevant to listeners’ perceptions of tempo (Bosker et al., 2017; Bosker & Reinisch, 2017), we conducted an online survey to get a quantitative measure of the “fittedness” of the crucial lexical items in their carrier sentences. We ran this survey using *Google Forms* (https://www.google.com/forms/). The survey included both members of each lexical pair. This generated 52 sentences. We added filler sentences from the set of additional sentences described above to make 80 sentences. The same carrier never occurred in consecutive sentences. Participants judged for each sentence how easy it was to think of a context in which the sentence makes perfect sense, with the response options “Easy,” “Quite hard,” and “Very hard,” which we converted to numerical scores 1, 2 and 3 respectively. Twenty-five native speakers of British English in the age range 18‒35 completed the survey. The results suggested that most sentences are reasonably well formed. Table 2 shows that only one sentence scored over 2.5, *I saw John’s polite*, which we changed to *I see John’s polite* (Sentence 23) to make it easier to interpret. Table 2 also shows the difference in goodness per sentence pair; this allows us to assess whether any observed effect of the imposed interpretation of ambiguous forms is modulated by the semantic goodness difference between the [+schwa] and [‒schwa] interpretations.

**Table 2.** Semantic goodness scores given [‒schwa] and [+schwa] sentence orthographies.

Structure	Sentence	[–schwa] orthography, *M* (*SD*)	[+schwa] orthography, *M* (*SD*)	Mean difference
fricative-(schwa)-plosive	1. He wanted to *sport~support* it.	1.56 (0.70)	1.00 (0.00)	–0.56
	2. He *sported~supported* that jumper.	1.12 (0.43)	2.08 (0.63)	0.96
	3. He listed *sporting~supporting* laws.	1.20 (0.40)	1.28 (0.53)	0.08
	4. He often *sports~supports* this.	1.32 (0.55)	1.08 (0.27)	–0.24
	*M*	1.3	1.36	0.06
voiced plosive-(schwa)-liquid	5. Why not *blow~below* the candles?	1.40 (0.75)	1.84 (0.78)	0.44
	6. They *dried~deride* it.	1.04 (0.20)	2.04 (0.77)	1.00
	7. The distance was *drivable~derivable*.	1.12 (0.43)	2.04 (0.72)	0.92
	8. They *drive~derive* it.	1.20 (0.40)	1.44 (0.64)	0.24
	9. This tool *drives~derives* it.	1.60 (0.69)	2.44 (0.64)	0.84
	10. We are *driving~deriving* the length.	1.72 (0.72)	1.84 (0.67)	0.12
	11. He spotted the *gorilla~griller*.	1.52 (0.64)	1.00 (0.00)	–0.52
	12. I counted five *gorillas~grillers*.	1.56 (0.57)	1.00 (0.00)	–0.56
	*M*	1.40	1.71	0.31
voiceless plosive-(schwa)-liquid	13. He predicted the *claps~collapse*.	1.52 (0.70)	1.08 (0.39)	–0.44
	14. It was *clean~Colleen* today.	1.12 (0.32)	1.60 (0.69)	0.48
	15. It was the first *clone~cologne*.	1.12 (0.43)	1.36 (0.48)	0.24
	16. I tried these *clones~colognes*.	1.96 (0.72)	1.08 (0.27)	–0.48
	17. They *clued~collude* in the police.	1.52 (0.75)	2.12 (0.71)	0.60
	18. It was that *cream~Kareem* again.	1.48 (0.64)	1.40 (0.63)	–0.08
	19. I love this *cress~caress*.	1.24 (0.51)	1.68 (0.68)	0.68
	20. They *crowed~corrode* on the roof.	1.56 (0.70)	1.48 (0.64)	–0.08
	21. He spotted the *Kroner~corona*.	1.48 (0.64)	1.16 (0.37)	0.04
	22. I counted 10 *Kroners~coronas*.	1.36 (0.48)	1.12 (0.43)	–0.24
	23. I see John’s *plight~polite*.^a^	1.36 (0.48)	2.80 (0.40)	1.44
	24. At the end we *parade~prayed*.	1.00 (0.00)	1.80 (0.69)	0.80
	25. I don’t like this *train~terrain*.	1.00 (0.00)	1.12 (0.32)	0.12
	26. I saw those *trains~terrains*.	1.00 (0.00)	1.76 (0.59)	0.76
	*M*	1.34	1.54	0.27
Overall *M*		1.35	1.56	0.25

a Scores are for the original version of this item, *I saw John’s plight~polite* (see text for details).

##### Recording

All sentences were produced by a female speaker of British English (age 27) who grew up in the South East of England. Recordings were made in a soundproof room using a cardioid condenser microphone (Audio-Technica AT2020), a USB audio interface (M-Audio Fast Track Pro) and the recording software *Audacity* running on a Windows PC. The recordings were produced at a sampling rate of 44100 Hz with 32-bit amplitude resolution and then exported as mono WAV files with 16-bit resolution. The speaker produced each sentence once at a normal pace and once at a fast pace with as little variation in pitch and loudness contours across sentences as feasible.

Impressionistic analysis of the speaker’s productions suggested that she regularly “deleted” /ə/ in [+schwa] words when speaking at fast pace: she realised words such as *derive* /dəˈɹaɪv/ without a clearly audible [ə]. She occasionally did this when speaking at a normal pace too. Of course, a lexical difference between, for example, *drive* and *derive* correlates with phonetic differences on multiple parameters, and some of these differences may remain observable even if a segment-size realisation of schwa is not (Coleman, 1992; Davidson, 2006). Listeners are able to orient to such fine phonetic detail (Desmeules-Trudel & Zamuner, 2019; Gow, 2003; Manuel, 1995), and one may, therefore, question whether in some or indeed many cases described in terms of “deletion,” acoustic cues for relevant phonological distinctions are really notably absent (Hawkins, 2003; Local, 1992).

Initial listening and a small-scale audio survey on 10 native British English speakers indicated that while the speaker’s productions were in a suitable style for our purposes, they were not sufficiently ambiguous to be compatible with both [+schwa] and [‒schwa] orthographies. Thus, we would need to manipulate them to create maximally ambiguous forms. We judged that the best starting point for further manipulations was the speaker’s normal-pace productions of the sentences, with the fast-pace production of [+schwa] words spliced in, for example, [*He wanted to*]_normal_ [*support*]_fast_ [*it*]_normal_. With a normal-pace carrier, listeners may not have a heightened expectation of syllable deletion (Koreman, 2006), and indeed, for a subset of these versions, survey results revealed that listeners accepted them as a good match for both [+schwa] (*He wanted to support it*) and [‒schwa] (*He wanted to sport it*) sentences. Thus, the further manipulations described below were applied to fast-pace [+schwa] tokens embedded in normal-pace carriers.

##### Manipulation

For the *plosive–schwa–liquid* words, we manipulated schwa duration and plosive VOT, as VOT tended to be longer when a schwa followed than when no schwa followed. Contrary to what might be expected on the basis of descriptions of English phonetics (Collins & Mees, 2013; Ogden, 2009), we did not find that the speaker consistently devoiced liquids in *plosive–liquid* words starting with voiceless plosives (*Kroner*, *cress*, *clean*, etc.). We, therefore, did not manipulate this parameter. For *support* and related words, we observed, as expected, that the VOT of /p/ was consistently longer in [–schwa] words than in [+schwa] words (see Ogden, 2009). We, therefore, manipulated [ə] duration and the VOT of /p/.

We segmented the relevant portions of the schwa words in *Praat* (Boersma & Weenink, 2017). VOT was delimited from the start of the first positive-going waveform deviation to the start of periodicity. The boundary between [ə] and a following liquid was placed at the point where waveform amplitude reached its minimum or formant frequencies reached their most extreme values for the liquid. This means that transitions into the liquid were included in the [ə] interval. (We opted for this method after finding that under the alternative—including the transitions in the liquid segment—these transitions gave rise to /ə/ percepts even when the entire [ə] segment was removed.) We extracted [ə] and VOT durations and calculated 25%, 50%, and 75% proportions for each, with a view to shortening the segments in several steps. We implemented the shortenings by removing signal portions manually from the middle of the segments, maintaining the transitions out of and into adjacent phones. For [ə], we ensured that the removed portions contained only complete periods of vibration, to maximise spectral continuity. In some cases, this meant that the removed portion was fractionally short or long.

Exploratory auditory analysis suggested that reducing [ə] duration by only small amounts left a clear [ə] percept while reducing VOT by a large amount made voiceless plosives sound voiced. We, therefore, produced a set of manipulated forms in which [ə] was zero or 25% of its original duration and VOT was 50%, 75% or 100%. This yielded six versions of each word and (6 × 26 =) 156 candidate experimental sentence forms. The three authors listened to all forms and judged for each whether it was hearable as both a [+schwa] and a [–schwa] word. We excluded from further consideration forms for which fewer than two of us deemed this to be the case. This reduced the size of the set to *N* = 97. We then conducted an online listening survey in which listeners judged how well the audio and written form of selected sentence forms matched. We ran this survey using *SoSci Survey* (www.soscisurvey.de), which allows for audio file embedding. We presented the 97 candidate experimental sentence forms without fillers and made the participants aware that they were judging multiple versions of each sentence. Participants were asked to focus on how well the audio matched the written sentence, rather than the goodness of the sentence in terms of grammar or meaning. Participants submitted their judgements using a slider which mapped to a 100-point scale.

To get two goodness ratings for each candidate ambiguous word form—one given a [–schwa] orthography and one given a [+schwa] one—we produced two versions of the survey for which orthography was counterbalanced across items. Multiple manipulations of the same sentence were transcribed the same within each survey version and were non-adjacent in the trial order. The survey was completed by 37 native speakers of British English in the age range 18‒45 who had completed neither of the surveys reported above; 18 completed one version, 19 the other. In addition to the two goodness ratings per sentence form, we calculated the difference between these, so we could identify the manipulations that yielded the most similarly acceptable sentence forms across the two orthographies. Where multiple manipulations yielded very similar difference values but rather different goodness values, we selected the manipulation that yielded the highest goodness values across the two orthographies. The optimal manipulations are given in Table 3 with their VOT and [ə] durations as a percentage of the original value, and their mean goodness ratings.

**Table 3.** Auditory goodness scores for the optimal manipulated sentence forms given [‒schwa] and [+schwa] sentence orthographies.

Structure	Sentence	VOT %	[ə] %	[–schwa] orthography, *M* (*SD*)	[+schwa] orthography, *M* (*SD*)	Mean difference
fricative-(schwa)-plosive	1. He wanted to *sport~support* it.	50	0	49.8 (35.1)	84.4 (34.6)	34.6
	2. He *sported~supported* that jumper.	100	0	35.4 (31.0)	88.8 (18.1)	53.4
	3. He listed *sporting~supporting* laws.	75	0	61.5 (32.3)	91.8 (12.9)	30.3
	4. He often *sports~supports* this.	50	0	42.3 (32.0)	79.1 (20.3)	36.8
	*M*			47.3	86.0	38.8
voiced plosive-(schwa)-liquid	5. Why not *blow~below* the candles?	50	25	85.8 (25.8)	58.3 (30.1)	–27.5
	6. They *dried~deride* it.	100	25	93.6 (9.1)	55.6 (32.9)	–38.0
	7. The distance was *drivable~derivable*.	100	0	89.3 (16.0)	29.0 (24.0)	–60.3
	8. They *drive~derive* it.	100	25	91.1 (13.3)	47.7 (25.2)	–43.4
	9. This tool *drives~derives* it.	100	0	90.2 (12.4)	46.1 (30.4)	–44.1
	10. We are *driving~deriving* the length.	100	0	88.7 (15.4)	37.2 (31.1)	–51.6
	11. He spotted the *gorilla~griller*.	100	0	87.1 (15.5)	79.4 (21.9)	–7.6
	12. I counted five *gorillas~grillers*.	75	0	69.6 (29.2)	64.7 (33.1)	–4.9
	*M*			86.9	52.2	–34.7
voiceless plosive-(schwa)-liquid	13. He predicted the *claps~collapse*.	75	0	70.9 (28.5)	89.1 (13.6)	18.2
	14. It was *clean~Colleen* today.	100	0	85.5 (21.0)	34.8 (22.4)	–50.6
	15. It was the first *clone~cologne*.	75	0	78.1 (25.6)	77.1 (21.9)	–1.0
	16. I tried these *clones~colognes*.	75	0	47.5 (29.2)	86.3 (18.6)	38.8
	17. They *clued~collude* in the police.	75	25	90.6 (11.6)	65.2 (31.2)	–25.5
	18. It was that *cream~Kareem* again.	100	0	93.6 (12.4)	76.1 (30.7)	–17.5
	19. I love this *cress~caress*.	100	25	91.3 (11.4)	62.5 (23.9)	–28.8
	20. They *crowed~corrode* on the roof.	100	25	83.3 (26.8)	85.6 (14.5)	2.3
	21. He spotted the *Kroner~corona*.	75	25	84.1 (18.2)	79.3 (29.4)	–4.9
	22. I counted 10 *Kroners~coronas*.	75	25	66.4 (25.7)	82.1 (20.9)	15.7
	23. I see John’s *plight~polite*.	100	0	69.7 (31.5)	67.8 (29.4)	–1.9
	24. At the end we *parade~prayed*.	100	25	83.1 (23.8)	38.8 (27.7)	–44.3
	25. I don’t like this *train~terrain*.	75	0	36.8 (30.8)	83.1 (19.6)	46.3
	26. I saw those *trains~terrains*.	100	25	51.8 (23.5)	67.2 (28.9)	15.3
	*M*			73.8	71.1	–2.7
Overall *M*				73.7	67.6	–6.2

Table shows that of the 26 selected sentence forms, 16 had goodness values above 50 given both orthographies, and difference values of at most 40. This means that participants were happy to accept the mapping between audio and transcription whether they were told the sentence contained a [+schwa] word or a [–schwa] word. In the remaining 10 sentence forms, one of the orthographies yielded an acceptability value below 50, and difference values were greater than 30. While we did not ask listeners to pay particular attention to any specific words in the sentences, we must accept that in these cases, our manipulations have not produced near-ambiguity. These 10 sentence forms did not appear to form a homogeneous subset: the relatively poor goodness values did not correlate with those of our semantic goodness survey; the crucial words had a range of phonological shapes; and in some cases, sentences with morphologically related word forms were apparently highly acceptable under both imposed interpretations. In the absence of clear leads for further acoustic parameters to manipulate, we decided to proceed with these 26 selected sentence forms.

We took several acoustic measurements over the 26 selected sentence forms, as experiments have shown that utterances with a relatively high *f*0 level, a relatively high magnitude of *f*0 movement and relatively high overall intensity are perceived as relatively fast (Feldstein & Bond, 1981; Kohler, 1986; Rietveld & Gussenhoven, 1987). Using *mausmooth* (Cangemi, 2015) in *Praat* (Boersma & Weenink, 2017), we extracted editable *f*0 contours for all of the sentences (time step of 0.05 s, analysis range 15–400 Hz) and manually removed clearly erroneous points. We then calculated the mean *f*0 for each corrected contour as a measure of *f*0 level and the kurtosis of the *f*0 distribution as a measure of span (Mennen et al., 2012; Niebuhr & Skarnitzl, 2019). We also took a mean intensity measure for each sentence form and recorded its duration.

#### Task

##### General design

To test the prediction that listeners who were orthographically prompted to interpret an *x* sentence form as [‒schwa] would rate it as slower than those who interpreted the same sentence form as [+schwa], we used a gradient implementation of an *abx* task in which participants rated the tempo of an *x* sentence form relative to two realisations of a second sentence: a slow realisation (*a*) and a fast realisation (*b*). Participants were shown a horizontal scale with the slow comparison sentence realisation (*a*) at the left end and the fast comparison sentence realisation (*b*) at the right. The participants’ task was to place the *x* sentence form on the scale according to its tempo relative to the comparison sentence realisations. The *a* and *b* sentence forms are similar to the “anchor sentences” used in some studies of speech tempo perception (Dellwo et al., 2006; Pfitzinger, 1999; Pfitzinger & Tamashima, 2006), except that they are different in each trial. The 26 sentences listed in Table 4 formed the crucial set of *x* sentences; the additional sentences without lexical ambiguity were used as comparison (*a* and *b*) sentence forms, as well as to construct filler trials.

**Table 4.** Articulation rates (syllables per second) for *x* sentences given a [‒schwa] orthography, and for *a*/*b* sentences by experimental block.

Structure	*x* sentence	*x* rate	*a*/*b* sentences (Block 1; Block 2)	*a–b* rates
fricative-(schwa)-plosive	1. He wanted to *sport~support* it.	6.42	It was that dream again. It was that canteen again.	5.78–7.06
	2. He *sported~supported* that jumper.	5.12	I counted five spillers. I counted five distillers.	4.61–5.63
	3. He listed *sporting~supporting* laws.	4.68	Why not join the candles? Why not conjoin the candles?	4.21–5.15
	4. He often *sports~supports* this.	4.44	They rode on the roof. They implode on the roof.	4.00–4.88
voiced plosive-(schwa)-fricative	5. Why not *blow~below* the candles?	5.45	We are proving the lot. We are improving the lot.	4.91–6.00
	6. They *dried~deride* it.	4.98	They grade it. They degrade it.	4.48–5.48
	7. The distance was *drivable~derivable*.	5.91	The sentence was delible. The sentence was indelible.	5.32–6.50
	8. They *drive~derive* it.	5.59	They fried it. They confine it.	5.03–6.15
	9. This tool *drives~derives* it.	4.9	I love this press. I love this success.	4.41–5.39
	10. We are *driving~deriving* the length.	5.75	He predicted the terms. He predicted the concerns.	5.18–6.33
	11. He spotted the *gorilla~griller*.	6.84	I counted 10 trainers. I counted 10 containers.	6.16–7.52
	12. I counted five *gorillas~grillers*.	5.31	He listed sorting laws. He listed consorting laws.	4.78–5.84
voiceless plosive-(schwa)-fricative	13. He predicted the *claps~collapse*.	5.56	They queued in the police. They intrude in the police.	5.00–6.12
	14. It was *clean~Colleen* today.	6.04	At the end we swayed. At the end we cascade.	5.44–6.64
	15. It was the first *clone~cologne*.	5.31	I don’t like this plane. I don’t like this champagne.	4.78–5.84
	16. I tried these *clones~colognes*.	4.13	I see John’s bright. I see John’s contrite.	3.72–4.54
	17. They *clued~collude* in the police.	6.01	He spotted the cleaner. He spotted the convenor.	5.41–6.61
	18. It was that *cream~Kareem* again.	6.24	He spotted the killer. He spotted the instiller.	5.62–6.86
	19. I love this *cress~caress*.	4.51	I tried these brooms. I tried these balloons.	4.06–4.96
	20. They *crowed~corrode* on the roof.	5.1	It was Greece today. It was Caprice today.	4.59–5.61
	21. He spotted the *Kroner~corona*.	6.46	He wanted to start it. He wanted to restart it.	5.81–7.11
	22. I counted 10 *Kroners~coronas*.	5.26	He needed that jumper. He preceded that jumper.	4.73–5.79
	23. I see John’s *plight~polite*.	5.02	I saw those cranes. I saw those campaigns.	4.52–5.52
	24. At the end we *parade~prayed*.	5.54	It was the first spoon. It was the first platoon.	4.99–6.09
	25. I don’t like this *train~terrain*.	5.13	He often courts this. He often retorts this.	4.62–5.64
	26. I saw those *trains~terrains*.	4.16	This tool seals it. This tool conceals it.	3.74–4.58
*M*		5.38		

We created two lists counterbalanced for orthography, so that all participants saw equal numbers of [+schwa] and [‒schwa] orthographic prompts, but only heard each sentence with one of the two orthographies. Within each group, participants got the same orthography for any morphologically related word forms. The lists were balanced for semantic and auditory goodness.

We presented participants with each *x* sentence twice, in two experimental blocks. The blocks differed in the nature of the *a/b* sentence forms. In Block 1, the *a/b* sentence consistently had the same number of syllables as the [‒schwa] interpretation of the *x* sentence. For example, the *x* sentence *He wanted to sport~support it* has six syllables in the [‒schwa] interpretation, so it was paired with *It was that dream again* which also has six syllables. In Block 2, the *a/b* sentence consistently had the same number of syllables as the [+schwa] interpretation of the *x* sentence. The *x* sentence *He wanted to sport~support it* has seven syllables in the [+schwa] interpretation, so it was paired with *It was that canteen again* which also has seven syllables. Therefore, if the shape of an *a/b* sentence form facilitated or hindered either the [‒schwa] or [+schwa] interpretation of the *x* sentence, it also did the opposite once in the course of the experiment. An *x* sentence was never paired with the *a/b* sentence that matched it structurally (e.g., *He wanted to {sport/support} it* was not paired with *He wanted to {start/restart} it*). The reason for the blocked design, and the fixed block order, was to avoid a situation where listeners heard, early in the experiment, many *a/b* forms containing a weak initial syllable (*restart*, *conjoin*, *success*, and so on). We were concerned that hearing numerous such forms might have created a type of structural priming that could bias listeners towards a [+schwa] interpretation of the ambiguous forms, making our orthographic manipulation less effective. We return to this point in the Discussion.

Each block consisted of 26 experimental trials and 14 filler trials. The same set of filler trials was used in both blocks. In seven filler trials, the *x* sentence was from the set of sentences that match the [‒schwa] experimental sentences in terms of phonological make-up (e.g., *He wanted to start it*) and the *a/b* sentence was from the set of sentences that match the [+schwa] experimental sentences (e.g., *He predicted the concerns*); in the other seven, it was the other way around.

##### Rate manipulations

To create the slow and fast realisations of the *a/b* sentence in the 52 experimental trials, we calculated the syllable rate for the *x* sentence form assuming a [‒schwa] interpretation. For example, *He wanted to {sport/support} it*, with duration 1.07 s, has a syllable rate of 1.07/6 = 6.42 syll/sec for the 6-syllable [‒schwa] interpretation. Next, we resynthesized the *a/b* sentence forms, to create versions at 0.9 (*a*) and 1.1 (*b*) of the *x* syllable rate. This was done separately for Block 1 (e.g., *It was that dream again*) and Block 2 (e.g., *It was that canteen again*). The resulting rates are given in Table 4. The resynthesis was done using PSOLA in Praat (Boersma & Weenink, 2017). Informal piloting suggested that the 10% rate adjustments were easily perceivable without resulting in extremely slow or fast realisations.

For the 14 filler trials, six filler sentence pairs were subjected to the same manipulation pattern, with the syllable rates of *a* and *b* set at 10% below and above the syllable rate of the *x* sentence form. Four filler pairs had the rate of *a* matching that of *x*, with that of *b* fixed at 20% faster, and four had the rate of *b* matching that of *x*, with that of *a* fixed at 20% slower. This design presented listeners with a degree of variety in the rate ranges across trials.

#### Procedure

**Figure 1.**
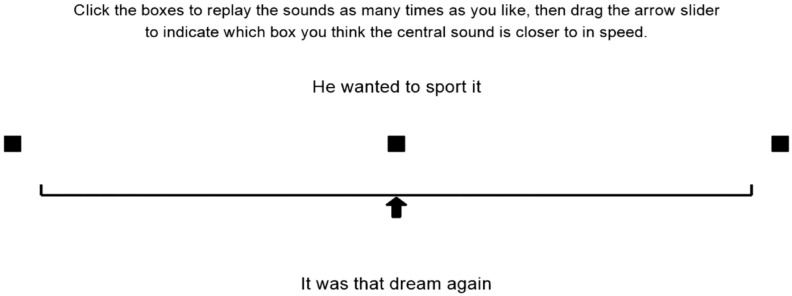
Visual interface for one experimental trial (Experiment 1).

The experiment interface was coded in *PsychoPy2* (Peirce, 2009). Each trial had the same structure, illustrated in Figure 1. First, the instructions and sliding scale appeared. Next, the *x*, *a*, and *b* stimuli were presented one at a time. In each case, both the orthography and a black square appeared on the screen and after 1.5 s the audio played. The first to appear was *x*, in the middle of the sliding scale. On alternating trials, the next to appear was *a* (the slower version, at the left scale end) or *b* (the faster version, at the right scale end). The orthography for the *x* sentence was displayed at the top of the screen, that for the *a/b* sentence at the bottom. The visual elements remained on screen for the duration of the trial and participants could replay any of the three audio stimuli.

To record their judgements of the relative tempo of the *x* stimulus, participants were asked to click and drag the corresponding square leftwards or rightwards along the scale from its central starting position. Once they had started dragging the *x* square, an arrow appeared underneath it to aid its positioning, and a “next screen” instruction appeared, allowing participants to advance to the next trial. This meant that they could not advance without moving the slider at all, although they were able to decide on a central position after doing so.

The experiment started with a short practice session comprising three filler trials. The 40 Block 1 trials and 40 Block 2 trials were then presented with an optional break after each 20 trials. Trials were presented in the same within-block randomised order for all participants. The randomised order was different between blocks. No qualitative feedback was elicited.

#### Quantitative analysis

To test our prediction that *x* sentences would be rated as slower when participants were orthographically prompted to interpret them as containing [‒schwa] than [+schwa] words, we fitted linear mixed effects models using *lme4* (Bates et al., 2015), *lmerTest* (Kuznetsova et al., 2017) and *emmeans* (Lenth, 2022) in *R* (R Development Core Team, 2008). We used a stepwise model fitting procedure, performing model comparisons using the *anova* function (Baayen, 2008). All factors were treatment coded. The dependent variable was the participants’ tempo ratings, henceforth Rating (*N* = 3,640). These were recorded on a numerical scale from 0 to 1000, with 500 representing a central placement of the stimuli in the visual interface. The crucial predictor variable was the orthography of the *x* sentence (henceforth *Orthography*): [‒schwa] or [+schwa]. Since our rating scale is bounded on both ends, we also fitted beta regression models using *glmmTMB* (Brooks et al., 2017). We followed the same stepwise modelling procedure as we did with the linear mixed effects models, and the two methods revealed the same data patterns and pointed to the same optimal model. In what follows, we will report the optimal linear mixed effects model only.

We included random intercepts for *Participant*) and *Item.* The latter distinguishes the *x* sentence forms irrespective of the imposed interpretation. Each level of *Item* is repeated within participants (as each *x* was presented in Blocks 1 and 2) and across participants (as each *x* was presented with a [‒schwa] or [+schwa] interpretation depending on the participant). Models with random slopes failed to converge.

As the experiment consisted of two blocks of trials and we varied the presentation order of the *a* and *b* sentence forms, we coded for *Block* (1 or 2), *Trial* (within blocks) and *Order* (*a* first or *b* first). We also included variables from the surveys we had run—*Semantic goodness* and *Manipulation goodness* per sentence form and *Semantic goodness difference* and *Manipulation goodness difference* across the corresponding [‒schwa] and [+schwa] sentences—and from acoustic analysis of the selected *x* sentence forms—*f0 mean*, *f0 kurtosis*, *Intensity mean*, and (log-transformed) *Duration*.

Prior to modelling, we inspected the relationship between our crucial predictor variable, *Orthography*, and our control variables by running simple linear models for the latter with *Orthography* as the only predictor and [‒schwa] the reference level. This revealed that *Orthography* is systematically related to both *Semantic goodness* (est = 0.214, se = 0.013, *t* = 16.95, *p* < .001) and *Manipulation goodness* (est = −5.567, *SE* = 0.617, *t* =−9.024, *p* < .001). The coefficients reflect that for *Semantic goodness*, sentences with [+schwa] words were rated as more acceptable (Table 2); for *Manipulation goodness*, sentences with [+schwa] words were rated as less acceptable (Table 3).

### Results

**Figure 2.**
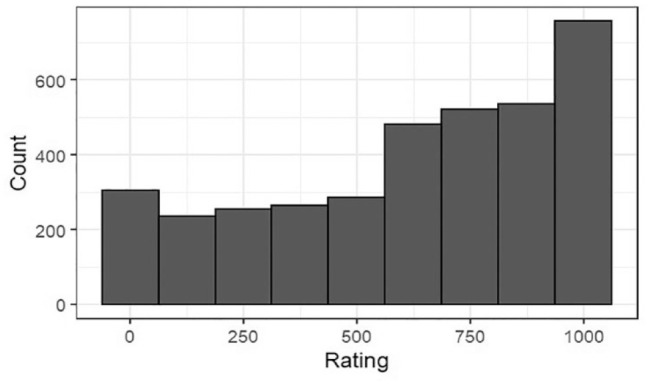
Distribution of *Rating* (*N* = 3,640).

As seen in Figure 2, the distribution of *Rating* is skewed towards participants judging *x* as closer in tempo to *b*. We fitted our models on the raw values of *Rating* and on the results of a square-root transformation—formula *sqrt, max(x* *+* *1, –x)*—which reduced the skewness. As the outcomes were the same, we present the modelling procedure using the raw values.

In modelling *Rating*, we started with a base model with random intercepts for *Participant* and *Item* and first assessed the predictive value of the design-related control variables *Block*, *Trial*, and *Order*. This revealed that adding *Block* to the model improved fit; adding *Trial* or *Order* did not. We then assessed the predictive value of *Orthography* along with *Semantic goodness* and *Manipulation goodness*. As indicated above, *Orthography* is systematically related to both acceptability variables, so if any of the three variables significantly improved model fit, the question was which resulted in the strongest model—operationalised as the model with the lowest AIC value (Baayen, 2008). *Orthography* turned out to be the strongest of the three variables, and with it added to the model neither *Semantic goodness* nor *Manipulation goodness* further improved fit. We then established that an interaction between *Block* and *Orthography* significantly improved fit while interactions between *Orthography* on one hand and *Semantic goodness difference* and *Manipulation goodness difference* on the other did not. Finally, we checked whether any of the acoustic variables further improved fit. Perhaps unsurprisingly, given the presence of *Item* as a random effect, we did not find evidence of this.

**Figure 3.**
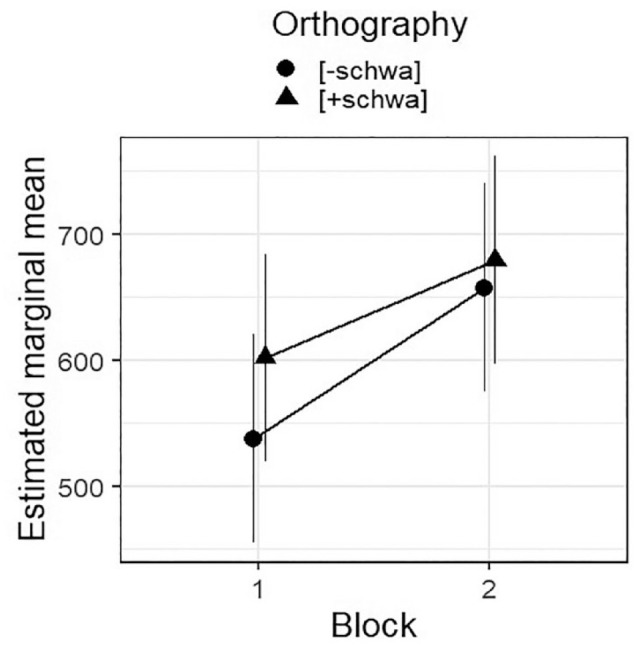
Estimated marginal means, with confidence intervals, for the combined variable levels of *Block* and *Orthography* in the optimal model for *Rating* (see Table 3).

The resulting optimal model is summarised in Table 5; Figure 3 plots the estimated means for all combined variable levels. (The random effects coefficients are: Participant variance = 2,383, *SD* = 48.81; *Item* variance = 39,744, *SD* = 199.36; residual variance = 59,957, *SD* = 244.86.) Listeners’ tempo ratings were significantly higher in Block 2 than in Block 1. They were also significantly higher for [+schwa] sentences than for [‒schwa] sentences, in line with our hypothesis. The significant interaction between *Block* and *Orthography* reflects that the effect of *Orthography* is strongly significant in Block 1, but misses significance at α = .05 in Block 2 (post hoc contrast: est = 21.9, *SE* = 11.5, *df* = 3,542, *t* = 1.912, *p* = .056). We will consider why this might be below.

**Table 5.** Summary of fixed effects in the optimal model of *Rating*. For *Block*, “1” is the reference level; for *Orthography*, “[‒schwa]” is the reference level.

	Estimate	*SE*	*df*	*T*	*p*
(Intercept)	537.55	40.36	27.77	13.32	<.001
*Block* “2”	119.82	11.48	3542	10.44	<.001
*Orthography* “[+schwa]”	64.15	11.48	3542	5.59	<.001
*Block* “2” × *Orthography* “[+schwa]”	–42.21	16.23	3542	–2.60	.009

### Discussion

The results of this experiment confirm our prediction that listeners who were orthographically prompted to interpret an *x* sentence as containing a [‒schwa] word (e.g., *sport*) would rate the sentence form as slower than those who were prompted to interpret it as containing the corresponding [+schwa] word (*support*): *Orthography* was a significant predictor of *Rating*, at least in Block 1, and in both blocks the [+schwa] orthography was associated with higher mean tempo estimates than the [‒schwa] orthography. This supports our hypothesis that listeners are influenced by the canonical form when judging tempo.

We noted that in our stimulus set, *Orthography* is systematically related to both *Semantic goodness* and *Manipulation goodness*. It is, therefore, possible that some of the observed effect of *Orthography* is really an effect of either of these acceptability measures: specifically, that [+schwa] sentences sounded faster because they were more semantically acceptable, and/or acoustically poorer exemplars. There is some support in the literature for the latter idea. Both cognitive load and an unfamiliar language increase perceived tempo, perhaps because listening effort results in sparser temporal sampling of the signal (Bosker, 2017; Bosker & Reinisch, 2017). If sentences with a poorer mapping of sound to acoustic form also require more listening effort, this could explain why they are heard faster. However, semantic acceptability patterns in the opposite direction for our stimuli, making an effort-based explanation less convincing overall. Moreover, on statistical grounds, it seems unlikely to us that the observed effect can be attributed entirely to effects of the goodness measures. First, *Orthography* was clearly the strongest predictor out of the three, that is, it explains variance in *Rating* that neither *Semantic goodness* nor *Manipulation goodness* does. Second, modelling the ratings for a stimulus subset which did not contain the collinearity (see Experiment 2 for more detail) yielded an optimal model with the same significant effects as those listed in Table 5. Thus, the orthographic manipulation itself remains the strongest explanation for the effect.

Why was the effect of *Orthography* only significant in Block 1? One possibility is that listeners’ attention to the orthographic prime waned over the course of the experiment. We did not obtain a measure of how much they looked at the orthographic sentence form, and it is possible that as the experiment went on, they chose to ignore it and answer based on the auditory stimulus alone. Such a response strategy, which can also be conceptualised as a type of fatigue effect, might, though, more likely have given rise to a *Trial* effect (which we did not find) than a *Block* effect.

An alternative explanation is based on the stimuli themselves. To understand this, we first note that ratings were higher overall, that is, closer to the faster (*b*) endpoint, in Block 2 than Block 1. Although we used the same rates for *a* and *b* in both blocks, the *a/b* sentences in Block 2 had one more syllable than those in Block 1, so their durations were longer in Block 2 than Block 1. Crucially, in Block 2, both *a* and *b* were systematically longer in duration then *x.* In data analysed after Experiment 1 was designed, Plug et al. (2022) observed that utterance duration affects tempo perception over and above effects of syllable rate, with longer utterances heard as slower than shorter ones. Applying the same logic here, the short duration of *x* sentences (relative to *a* and *b*) in Block 2 may explain why *x* was rated overall as closer to *b* (the faster endpoint) in Block 2. Against this context, the *Block × Orthography* interaction may simply indicate that any potential effect of orthography in Block 2 was washed out by the overall higher perceived tempo of the *x* stimuli.

A final possibility is that a type of structural priming occurred in Block 2. In this block, all *a/b* sentences contained a weak syllable. Listeners may have noticed the systematic presence of this additional syllable, especially since they had heard its comparator in Block 1 (e.g., Block 1 *It was that dream again*; Block 2 *It was that canteen again*), and this may have made the [+schwa] interpretation of *x* sentences difficult to ignore, with the result that the *Orthography* manipulation was simply less effective in Block 2. Recall that avoiding structural priming of this kind was the reason we selected the blocked design in the first place; however, counterbalancing block order or indeed a fully randomised design might have been a better solution.

Finally, while in line with our hypothesis for Block 1 at least, we note that the effect of *Orthography* was relatively small. Even in Block 1, the average response difference between [+schwa] and [‒schwa] interpretations was only 64 points on the 1000-point scale. Given the small size of the effect, we wanted to test whether it would survive if participants had to make more categorical judgements of relative tempo. In designing Experiment 2, we also avoided the block structure of Experiment 1 and reduced the stimulus set to ensure there was no significant relationship between *Orthography* on one hand and *Semantic goodness* and *Manipulation goodness* on the other.

## Experiment 2

### Method

#### Participants

Eighty-two native speakers of British English (48 females) in the age range 18–35 (*M* = 24) participated in this experiment. All self-reported as having grown up in a monolingual household and having no known hearing problems. All provided informed written consent in line with institutional ethics clearance (University of Leeds, Faculty of Arts, Humanities and Cultures Ethics Committee, LTSLCS-072). None had participated in Experiment 1. All were paid for their time.

#### Stimulus set

We noted above that in the full set of 26 sentences, *Orthography* is systematically related to both *Semantic goodness* and *Manipulation goodness*. To remove this collinearity for Experiment 2, we plotted these variables and removed data points until the remaining clouds showed no significant correlations. This left a set of 15 sentences (Table 6).

**Table 6.** Stimulus subset used in Experiment 2, with auditory and semantic goodness scores.

Structure	Sentence	Auditory goodness, *M* (*SD*)		Semantic goodness, *M* (*SD*)	
		[–schwa]	[+schwa]	[–schwa]	[+schwa]
fricative-(schwa)-plosive	He wanted to *sport~support* it.	49.8 (35.1)	84.4 (34.6)	1.56 (0.70)	1.00 (0.00)
	He often *sports~supports* this.	42.3 (32.0)	79.1 (20.3)	1.32 (0.55)	1.08 (0.27)
voiced-plosive-(schwa)-liquid	We are *driving~deriving* the length.	88.7 (15.4)	37.2 (31.1)	1.72 (0.72)	1.84 (0.67)
	He spotted the *gorilla~griller*.	87.1 (15.5)	79.4 (21.9)	1.52 (0.64)	1.00 (0.00)
	I counted five *gorillas~grillers*.	69.6 (29.2)	64.7 (33.1)	1.56 (0.57)	1.00 (0.00)
voiceless-plosive-(schwa)-liquid	He predicted the *claps~collapse*.	70.9 (28.5)	89.1 (13.6)	1.52 (0.70)	1.08 (0.39)
	It was *clean~Colleen* today.	85.5 (21.0)	34.8 (22.4)	1.12 (0.32)	1.60 (0.69)
	It was the first *clone~cologne*.	78.1 (25.6)	77.1 (21.9)	1.12 (0.43)	1.36 (0.48)
	I tried these *clones~colognes*.	47.5 (29.2)	86.3 (18.6)	1.96 (0.72)	1.08 (0.27)
	They *crowed~corrode* on the roof.	83.3 (26.8)	85.6 (14.5)	1.56 (0.70)	1.48 (0.64)
	He spotted the *Kroner~corona*.	84.1 (18.2)	79.3 (29.4)	1.48 (0.64)	1.16 (0.37)
	I counted 10 *Kroners~coronas*.	66.4 (25.7)	82.1 (20.9)	1.36 (0.48)	1.12 (0.43)
	At the end we *parade~prayed*.	83.1 (23.8)	38.8 (27.7)	1.00 (0.00)	1.80 (0.69)
	I don’t like this *train~terrain*.	36.8 (30.8)	83.1 (19.6)	1.00 (0.00)	1.12 (0.32)
	I saw those *trains~terrains*.	51.8 (23.5)	67.2 (28.9)	1.00 (0.00)	1.76 (0.59)
Overall *M*		68.33	71.21	1.39	1.30

#### Task design

We used a pairwise comparison task: participants heard pairs of sentences and had to decide for each pair whether the second pair member sounded slower than, faster than or the same as the first pair member. This paradigm has been used in multiple previous studies on speech tempo perception (Plug & Smith, 2021; Quené, 2007; Weirich & Simpson, 2014). The 15 sentences containing an ambiguous word form (*claps*~*collapse* and so on) formed the crucial set of *x* sentences. Each was paired with a *y* sentence which contains no ambiguity and has the same number of syllables as the [–schwa] interpretation of the *x* sentence. For example, *He predicted the claps~collapse*, which has six syllables with *claps* and seven with *collapse*, was paired with *They implode on the roof* with six syllables. As in Block 1 of Experiment 1, we only used sentences with [–schwa] comparison words for the *y* sentences.

Using PSOLA in Praat as before, we manipulated the duration of each *y* sentence to be identical to that of its *x* sentence. This means that on a [–schwa] interpretation of *x*, *x* and *y* have the same syllable rate; on a [+schwa] interpretation of *x*, *x* has a higher syllable rate than *y*. We presented all pairs in two orders: *xy* and *yx*. In all cases, participants were asked to assess how the last pair member they heard—*y* in *xy* pairs, *x* in *yx* pairs—compared with the first in tempo. We predicted that when presented with a [+schwa] orthography for *x*, participants would rate *x* as faster than *y* (or *y* as slower than *x*) more often than when presented with a [–schwa] orthography for *x*.

We constructed 12 filler pairs. In each, the two sentences had the same number of syllables (four, five, six or seven): for example, *They rode on the roof ~ I love this success* with five syllables. For four filler pairs, the duration of one pair member was manipulated to match the (unmanipulated) duration of the other, so that both pair members had the same syllable rate. For eight pairs, the duration of one pair member was set at 0.95 of the other’s (unmanipulated) duration, so that one pair member has a higher syllable rate and should be audibly faster. Like the experimental sentence pairs, the filler pairs were presented in both orders.

This method results in 54 trials: 15 experimental sentence pairs presented in two orders plus 12 filler pairs presented in two orders. To maximise response numbers, we presented these 54 trials twice, in two experimental blocks separated by an optional break (108 trials in total). The only difference between the blocks was in the trial order, which was set to random in each block for each participant.

#### Procedure

As in Experiment 1, we created two counterbalanced lists, such that each sentence was presented with [+schwa] and [‒schwa] orthography to different groups of participants, and all participants were exposed to approximately 50% [+schwa] and 50% [‒schwa] orthographies, and never to both orthographies for the same sentence.

**Figure 4.**
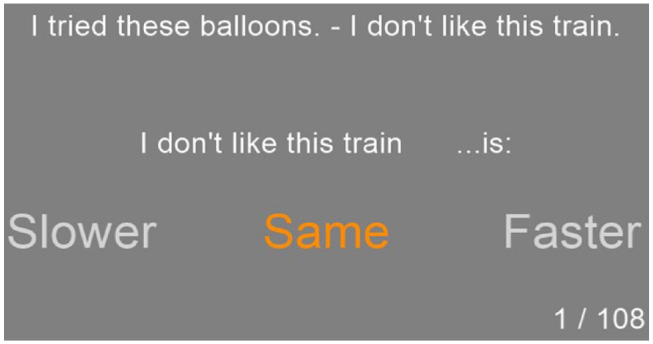
Visual interface for one experimental trial (Experiment 2).

The experiment interface was again coded in *PsychoPy2* (Peirce, 2009). Each trial had the same structure, illustrated in Figure 4. First, the sentence pair was displayed on the screen. After 5 s of reading time, the corresponding audio played. Immediately after the audio had played, the second sentence’s orthography was repeated followed by “is” and, centred underneath in larger font size, “Slower,” “Same,” and “Faster.” Participants were asked to record their judgements by clicking on the appropriate option, which changed colour when their cursor hovered over it. As soon as participants clicked, the next trial would start. Participants were not given the option to re-listen to any of the pairs. Before the main experiment, participants got four practice trials.

#### Quantitative analysis

To test our prediction that participants who were orthographically prompted to interpret an *x* sentence as containing a [+schwa] word would rate *x* as faster than *y*, we recoded responses to indicate whether each *x* was judged slower, same or faster than its corresponding *y.* We treated *Response* (“slow,” “same,” and “fast”) as an ordinal variable (Bürkner & Vuorre, 2019) and fitted cumulative link mixed models using the *ordinal* package (Christensen, 2018), *ggeffects* (Lüdecke, 2018) and *emmeans* (Lenth, 2022). As a control procedure, we also recoded *Response* as a numerical variable (“slow” = 1, “same” = 2, and “fast” = 3) and fitted linear mixed effects models using *lme4* (Bates et al., 2015), *lmerTest* (Kuznetsova et al., 2017), and *emmeans* (Lenth, 2022). In both cases, we used the same stepwise modelling approach as in Experiment 1 analysis. The two procedures revealed the same data patterns and pointed to optimal models containing the same significant effects. In what follows we will report the optimal cumulative link model.

We included random effects for *Participant* and *Item.* As for Experiment 1, the latter distinguishes the *x* sentence forms irrespective of the imposed interpretation. Each level of *Item* is repeated within participants (as each *x* was presented twice in an *xy* pair and twice in a *yx* pair) and across participants (as each *x* was presented with a [‒schwa] or [+schwa] interpretation depending on the participant). Models with random slopes failed to converge.

The crucial predictor variable was again the orthography of the *x* sentence (*Orthography*): [‒schwa] or [+schwa]. The position of the *x* sentence was coded as *Order* (*xy* or *yx*). As the experiment consisted of two sets of identical trials presented in a different order, we also used *Repetition* (1 or 2) and *Trial* (within repetition sets) as predictors. Exploratory modelling indicated that none of the additional variables from the surveys we had run—*Semantic goodness*, *Manipulation goodness*, *Semantic goodness difference*, *Manipulation goodness difference*—and from acoustic analysis of the *x* sentence forms—*f0 mean*, *f0 kurtosis*, *Intensity mean*, *Duration—*had explanatory value when *Item* was in the random effects; we, therefore, leave them aside in what follows.

### Results

**Figure 5.**
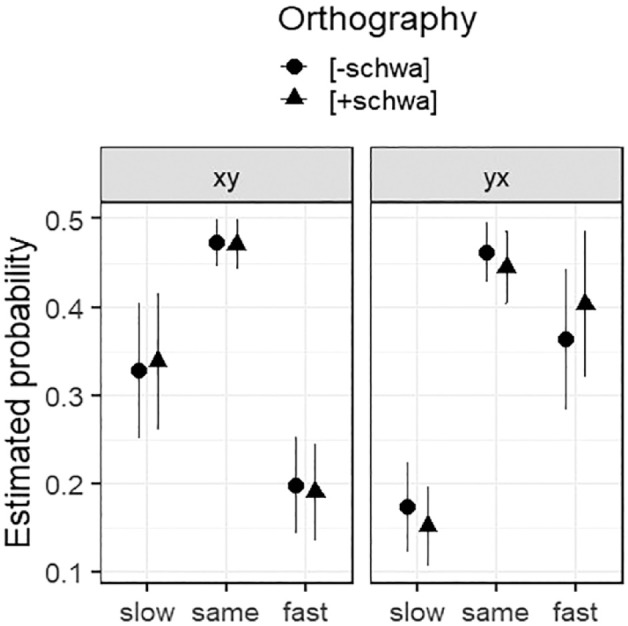
Estimated probabilities, with confidence intervals, for the combined variable levels of *Order* and *Orthography* in the optimal model for *Response* (see Table 7).

We started with a base model (logit link, flexible threshold) with random intercepts for *Participant* and *Item*. We first assessed the predictive value of the design-related control variables *Repetition*, *Trial*, and *Order*. This revealed that adding *Repetition* and *Order* to the model improved fit; adding *Trial* did not. We added *Orthography* as a main effect, and interactions of *Orthography* with *Order* and *Repetition*; the interaction with *Order*, but not that with *Repetition*, improved model fit. The resulting optimal model is summarised in Table 7. (The random effects coefficients are: *Participant* variance = 0.017, *SD* = 0.13; *Item* variance = 0.442, *SD* = 0.66.) The main effect of *Order* indicates that *x* was more likely to be judged faster than *y* when in *yx* sentences than *xy* sentences, that is, when *x* was heard last. The main effect of *Repetition* indicates that *x* was less likely to be judged faster in the second (repeated) set of trials. *Orthography* interacted with *Order*: while *Orthography* had no significant effect in *xy* pairs, in *yx* pairs it had the predicted effect, with *x* significantly more likely to be judged faster in [+schwa] sentences (post hoc contrast: est = 0.167, *SE* = 0.08, *z* = 2.17, *p* = .030). The effect in *yx* pairs is visible in Figure 5 in the raised estimated probability of “fast” for [+schwa]; the estimated probabilities of “slow” and “same” are reduced correspondingly, by similar degrees.

**Table 7.** Summary of fixed effects in the optimal model of *Response*. For *Repetition*, “1” is the reference level; for *Order*, “xy” is the reference level; for *Orthography*, “[‒schwa]” is the reference level.

	Estimate	*SE*	*z*	*p*
*Repetition* “2”	–0.128	0.05	–2.39	0.015
*Order* “yx”	0.837	0.08	10.81	<.001
*Orthography* “[+schwa]”	–0.049	0.07	–0.66	.508
*Order* “yx” × *Orthography* “[+schwa]”	0.216	0.11	2.02	.043

### Discussion

In interpreting the Experiment 1 results, we wondered whether the observed small effect of *Orthography* would survive if participants were asked to make more categorical judgements of relative tempo. In Experiment 2, participants made ordinal judgements rather than scalar ones in a pairwise comparison task. In designing the experiment, we also removed the possible confound between *Orthography* on one hand and *Semantic goodness* and *Manipulation goodness* on the other, and we avoided a structure in which experiment blocks involved qualitatively different judgements.

The effect of *Orthography* observed in Experiment 1 did indeed survive in this design, but only in stimulus pairs in which the manipulated sentence—the sentence with a lexically ambiguous word form for which the orthographic representation varied between participants—occurred last. For these stimulus pairs, *Orthography* was a significant predictor of *Response* such that [+schwa] sentences were more often heard as fast relative to their comparison sentence than [‒schwa] words. The effect again appears to be a small one, and occurred only for a subset of stimuli, a point to which we return in the General Discussion.

## General discussion

In this research, we assessed the robustness of “canonical form orientation” in speech tempo perception. Koreman (2006) and Plug et al. (2022) showed that listeners hear tempo differences between stimuli that are similar in surface articulation rates but different in canonical ones due to phone deletions. In their experimental designs, where stimuli were sampled from corpora of unscripted speech, the deletions co-occurred with other instances of phonetic reduction, and the tempo ratings could have derived from listeners’ recognition of a casual production style which they associated with fast speech. We asked whether their results can be replicated in an experimental design which minimises listeners’ ability to distinguish stimuli in terms of their overall production style. We imposed [‒schwa] and [+schwa] interpretations of ambiguous word forms through displayed orthography, and minimised differences between utterances by tightly controlling speaker and style. In both experiments, this manipulation yielded significant differences in response patterns in line with those reported by Koreman (2006) and Plug et al. (2022): [+schwa] interpretations were associated with higher tempo estimates than [‒schwa] interpretations. On the face of it, then, we can conclude that our results provide further evidence for listeners’ orientation to canonical forms when judging the tempo of spoken utterances.

This interpretation of our results is supported by a comparison of our study with that of Dilley and Pitt (2010). They report that phonetically reduced function words such as *or* fail to be recognised in potentially ambiguous segmental contexts when the articulation rate of a preceding phrase is low. For example, in *leisure or time* listeners associate a relatively long [ɹ] with *leisure* alone when the context articulation is slow—so interpret the phrase as *leisure time*. In a fast context, listeners hear the same phrase with the same relatively long [ɹ] as *leisure or time*. In Dilley and Pitt’s experiments, manipulations of local speech tempo affect listeners’ interpretations of phone strings that are close to lexically ambiguous. In our experiments, imposed interpretations of phone strings that are close to lexically ambiguous affect listeners’ perceptions of local speech tempo. It seems likely that both effects are the result of the same processing mechanism which maps an auditory signal to lexical representations. In explaining their findings, Dilley and Pitt (2010) argue that listeners estimate which of the two candidate articulations is most realistic given the context rate and the size of the critical time window. By analogy, in our experiments the [+schwa] orthographical representations may have prompted listeners to orient more to what they consider the “expected” articulations of [+schwa] words than to the acoustic signal—that is, to full or close-to-full pronunciation forms—and map those articulations to their observed time window to estimate speech tempo.

There are a number of reasons, however, to be cautious in interpreting our results. First, we cannot completely rule out that participants perceived variation in production style as a result of our orthography manipulation: when exposed to a [+schwa] sentence, the observation of schwa deletion alone may have led them to interpret the sentence production as relatively “casual”—and therefore, in some cases, relatively fast. We believe that our single-speaker design minimises the plausibility of this account even if it cannot be fully rejected. We should also note that it is debatable how exactly listeners apply their knowledge of associations between speech style and tempo in judging the tempo of new stimuli. Bosker et al. (2017) account for the finding that naturally fast speech is perceived as faster than linearly compressed speech with the same measured articulation rate (Reinisch, 2016) in terms of processing effort. On this view, speech with deletions is perceived as relatively fast not because deleted phones and syllables are perceptually “restored” and counted in a tempo estimation, nor because listeners recognise the naturally fast speech as more “casual” and therefore faster—but rather because mapping the reduced signal to lexical representations entails greater effort on the part of the listener than mapping a canonical realisation would (Janse et al., 2007; Ranbom & Connine, 2007). Several studies have indeed shown that increased cognitive load makes speech sound relatively fast, possibly due to a “shrinking of time” mechanism whereby additional attentional demands result in sparser temporal sampling of the speech signal (Bosker, 2017; Bosker et al., 2017; Bosker & Reinisch, 2017). An account along these lines would not appeal directly to listeners mapping canonical forms to their observed time windows in estimating speech tempo, but it would provide further support for the notion that canonical forms have a different status in tempo perception compared with reduced forms.

A second reason to be cautious in interpreting our results is that it is possible that the observed effects of our orthography manipulation are grounded more in how participants map acoustic input to orthographic forms than in their activation of full pronunciation forms: [+schwa] orthography typically has more letters than [‒schwa] orthography, and this may have informed participants’ tempo estimates. There is certainly ample evidence that “[p]art of what a listener knows about a word is how it is spelled” (Cutler & Davis, 2012, p. 1) and this knowledge can affect listeners’ performance in a range of tasks which are expected to activate phonological representations such as phoneme addition, deletion, and discrimination (Mann & Wimmer, 2002; Nayernia et al., 2019). We should note, however, that some of our [+schwa] words are associated with a grapheme string of the same length as that of their corresponding [‒schwa] word: for example, *griller*~*gorilla*, *prayed*~*parade*, *kroner*~*corona*. The other [+schwa] words are between one and three graphemes longer than their corresponding [‒schwa] word (*M* = 1.2). Explorative modelling reveals no evidence that relevant variables (difference vs no difference, or number of graphemes difference) have explanatory value.

Moreover, even if some of the observed effects can be attributed to our orthography manipulation *per se*, it is difficult to see how this could be avoided in an experimental design, given that “orthographic interference” has been observed even in “tasks that do not directly involve the printed word but are completed exclusively by listening or speaking” (Saletta et al., 2016). Saletta et al. report that pseudo-words that map transparently to one written form are repeated more quickly and accurately than those which map to multiple potential spellings, even if participants are not asked to consider the pseudo-words’ orthography. Similarly, Cutler and Davis (2012, pp. 5–6) note that “The task of rating the goodness of non-standard phoneme realizations requires no recourse to higher-level information, but its performance can be affected by orthographic knowledge when the phoneme realizations are embedded in real-word carriers.” In their study of the “suffix restoration effect,” whereby Dutch listeners reported hearing phonemes that were entirely absent from the acoustic signal (but present in corresponding canonical forms), Kemps et al. (2004) were able to tease apart effects of the orthographic and phonological representations, as one of the suffixes had two potential spellings: listeners could, therefore, be asked to monitor for a sound whose orthographical representation was not part of that of the host word. This revealed that listeners still reported hearing the sound. In the case of our experimental design, such disambiguation does not seem feasible. Given the nature of the lexical items we have to work with, replacing our orthographic stimuli by pictures does not seem straightforward either—and remarkably, even picture description tasks have been found to activate orthographic representations as much as phonological ones (Coch, 2018).

A third reason to be cautious in interpreting our results is that in both experiments the effects of the orthographic manipulation were small while additional variables related to the experimental design had larger effects on responses which also interacted with the orthography manipulation. Regarding the overall small size of the effect, this is perhaps not surprising given that [+schwa] words contain only one weak syllable, indeed only a single reduced vowel, more than their corresponding [–schwa] words. Severijnen et al. (2023) confirm that the relationship between syllable rate and perceived tempo increases is not straightforwardly linear, and suggest, among other things, that “unstressed syllables carry less weight than stressed syllables in computing speech rate.” Future work should test this hypothesis. Regarding the effects of the additional design variables, while the details differ across the two experiments, the pattern from both experiments raises interesting considerations regarding the rather fleeting and fragile nature of the effect of our orthographic manipulation.

In Experiment 1, we presented stimuli in two blocks which differed in the choice of comparison (*a/b*) sentences. We found a strong block effect, such that tempo estimates were generally higher in Block 2, and the effect of our orthography manipulation was only significant in Block 1. In Experiment 2, participants judged stimulus pairs, and we observed, as others have done (Lehiste, 1979; Plug & Smith, 2021; Weirich & Simpson, 2014), that participants tended to hear the second pair member as faster when they perceived a tempo difference. The effect of our orthography manipulation was only significant for pairs in which the *x* sentence was the second pair member. This means that in both experiments, the effect of our manipulation of orthography was observed for a subset of stimuli only.

In the case of Experiment 1, we speculated that the block effect might be due to the longer durations of the *a/b* sentences in Block 2 and the interaction might derive from a type of structural priming: the *a/b* sentences could be said to facilitate “schwa restoration” through priming, as they contained words like *concerns*, *preceded*, *contrite*, and so on, which in the context of the evolution of the stimuli and task may have meant listeners found it difficult to ignore a [+schwa] interpretation even on trials where [‒schwa] orthography was presented.

In the case of Experiment 2, the observation across multiple studies with similar designs of a listener bias towards hearing the second member of a pair of utterances as relatively fast warrants more detailed consideration. It seems plausible that in the context of a pairwise comparison task, short-term memory processes constrain listeners’ responses, as “any discrimination task necessarily entails that one stimulus is maintained in memory and compared with an incoming stimulus” (Mitterer & Mattys, 2017, p. 350). It is possible, therefore, that listeners’ attempt to keep a first pair member in memory while listening to the second results in a relative underestimation of the duration of the second pair member—and a relative overestimation of its speech tempo. This account does not explain why a tendency towards hearing the *x* sentence as relatively fast was observed in Experiment 1 too, as in Experiment 1, the *x* sentence was played before the *a* and *b* sentence forms. However, in Experiment 1 participants were allowed to replay any of the sentence forms. Unfortunately, the experimental data do not contain a record of replays, so we cannot confirm that participants made regular use of this option. With reference to a pairwise comparison task, one would predict that repeated exposure to the pairs would attenuate a cognitive load effect. This can be tested in a future experiment.

Short-term memory constraints may also underpin Experiment 2’s data pattern, where our orthography manipulation was only effective when the stimulus containing it was heard last. The literature on rate normalisation (see Bosker, 2017 for a review) distinguishes early temporal processing (which triggers some rate normalisation effects) from later processing which incorporates “higher level” information (and triggers other rate normalisation effects). Notably, Pitt et al. (2016) showed that the “lexical rate effect” arises only in intelligible speech contexts: unlike other rate effects in speech perception, such as the shifting of consonant voicing and manner boundaries in different rate contexts (Gordon, 1988; Wade & Holt, 2005), the effect observed by Dilley and Pitt (2010) cannot be primed by pure tone sequences or unintelligible sinewave speech. According to Bosker (2017), this suggests that the “lexical rate effect” operates at a later processing stage than rate effects that seem grounded in fast signal-based analysis. The application to the present findings is that under working memory constraints, the result of the early processing, which derives from basic signal-based beat tracking, may be retained better than the result of the later processing, which implicates top-down lexical and contextual knowledge. This would explain why the effect of our orthography manipulation is fleeting, such that it is not observed when another stimulus intervenes between the lexically ambiguous one and the listener’s response. Future work can test these speculations directly. Moreover, it is also worth exploring how much listeners actually attend to the presented orthography, for example, with eye-tracking.

We showed a small effect of deletions in an explicit tempo perception task; by contrast, Reinisch (2016) found that deletions affected rate perception in an implicit tempo judgement task, but had no measurable effect in an explicit task. Steffman and Jun (2021) observe a similar dissociation between explicit and implicit tasks, and speculate that explicit tasks may encourage “veridical” estimates of rate which draw more on physical duration than more cognitively influenced factors. Our results do not support this speculation. In consequence, it would be interesting to compare both types of task directly. While we opted against such a comparison for an initial approach to the question, it would seem possible to construct materials suitable for an implicit task: for example, using *I saw those trains~terrains on my right~ride* listeners would judge the identity of the final word after seeing one or other orthographic sentence prime. Another approach would be to follow Severijnen et al. (2023) in using word lists as the rate-setting context in an implicit task: listeners hear lists of ambiguous stimuli which they are primed to interpret as monosyllabic or disyllabic respectively *(train, sport, clone* versus *terrain, support, cologne*) before doing the phonetic categorisation of, for example, *right~ride.* Comparison of both tasks would shed further light on the level(s) and timescale(s) of processing over which tempo perception takes effect.

What are the broader implications for speech perception and processing, of an orientation to canonical form? Canonical forms have been argued to have a special status in speech perception (Brouwer et al., 2010; Bürki, 2018; Janse et al., 2007; Ranbom & Connine, 2007). Arguably, our finding of orientation to canonical form supports this special status: we have shown that, given a suitable context, even relatively poor-quality manipulated exemplars of reduced speech tokens can yield sufficient access to the canonical form to allow the canonical form to influence the listener’s impression of tempo (cf. Kemps et al., 2004). An emphasis on special status for canonical forms has tended to be associated with abstractionist accounts of speech perception (e.g., Norris, 1994; Norris & McQueen, 2008), in which variable signals are normalised to access lexical representations that themselves consist of fully specified canonical forms. However, taking our findings together with previous work showing influences of *both* canonical *and* surface form on tempo perception (Plug et al., 2022), we favour a more nuanced account. Experiments show that acoustic traces of the canonical form often persist in natural reduced speech, and that listeners are sensitive to them (Davidson, 2006; Ernestus & Smith, 2018; Manuel, 1995; Torreira & Ernestus, 2011; Warner & Tucker, 2011). If an exemplar is both relatable to a given canonical form (by virtue of its phonetic detail) and deviant from it, it can offer rich predictive information both about word identity and about the larger structure of which a word is part, including aspects of utterance rate and rhythmic structure which can inform subsequent perceptual decisions about segmental identity, location of word boundaries, and so on. On this view, the canonical form is not so much the obligatory target of lexical access, as a valuable reference point around which listeners can parse both the linguistic structure and socio-indexical properties of an utterance: see Warner et al. (2022) for a similar view. We would predict that the tempo information that can be accessed from naturally occurring reduced forms should be more robust than that obtained from manipulated forms such as those in our experiment.

## Conclusion

In summary, by leading listeners to believe that an ambiguous sentence form contains a word either with or without an additional schwa sound, we were able to shift their tempo percepts by small amounts in a direction consistent with the presence or absence of this additional schwa and syllable. It would seem clear from our findings, and those of Koreman (2006), Plug et al. (2022) and Reinisch (2016) that when listeners are asked to make tempo judgements, their responses do not reflect the outcome of fast signal-based analysis only, nor are they likely to reflect recognition of a casual speech style which listeners associate with fast tempo. Rather, the orientation to canonical form that has been observed across our and others’ studies reflects the complexity of influences on tempo perception, including both local and global aspects of the signal itself, its linguistic structure, and the listener’s interpretation of it.
